# Prognostic value of systemic inflammatory markers and the nutrition status in thymic epithelial tumors with complete resection

**DOI:** 10.1111/1759-7714.14485

**Published:** 2022-06-18

**Authors:** Tadashi Sakane, Katsuhiro Okuda, Takuya Matsui, Risa Oda, Tsutomu Tatematsu, Keisuke Yokota, Ryoichi Nakanishi

**Affiliations:** ^1^ Department of Oncology, Immunology and Surgery Nagoya City University Graduate School of Medical Sciences Nagoya Japan

**Keywords:** monocyte‐to‐lymphocyte ratio, neutrophil‐to‐lymphocyte ratio, prognostic nutritional index, thymic epithelial tumor

## Abstract

**Background:**

Recent studies have shown that several systemic inflammatory markers and the nutrition status, including the neutrophil‐to‐lymphocyte ratio (NLR), monocyte‐to‐lymphocyte ratio (MLR), platelet‐to‐lymphocyte ratio (PLR), and prognostic nutritional index (PNI), are useful prognostic factors in several malignant tumors. The present study explored the prognostic value of the NLR, MLR, PLR, and PNI in thymic epithelial tumor (TET) patients who underwent complete resection.

**Methods:**

A total of 158 TET patients who underwent complete resection were involved in the analysis. Their NLR, MLR, PLR, and PNI values were obtained from a blood examination within one month before the initiation of treatment. A receiver operating characteristic curve analysis was conducted to determine the optimal cutoff values.

**Results:**

The enrolled patients were stratified by cutoffs of 4.35 for the NLR, 0.22 for the MLR, 130.18 for the PLR, and 44.02 for the PNI. A univariate analysis revealed that high‐grade malignant TET, including type B2 and B3 thymoma, thymic carcinoma, and thymic neuroendocrine tumor; an advanced Masaoka stage; a high NLR; a high MLR; and a low PNI were significant predictors of a poor disease‐free survival (DFS). A multivariate analysis confirmed that an advanced Masaoka stage (HR = 5.5557, *p* = 0.0007) and a high MLR (HR = 3.3371, *p* = 0.0264) were independent predictors of a poor DFS.

**Conclusions:**

Our study demonstrated that the pretreatment MLR was an independent predictor of the DFS in patients with TETs who underwent complete resection.

## Graphical abstract

Kaplan–Meier curves for disease free survival according to the level of neutrophil‐to‐lymphocyte ratio (NLR) (a), monocyte‐to‐lymphocyte ratio (MLR) (b), platelet‐to‐lymphocyte ratio (PLR) (c), and prognostic nutritional index (PNI) (d).

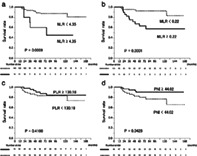

## INTRODUCTION

Thymic epithelial tumors (TETs), including thymomas, thymic carcinomas, and thymic neuroendocrine tumors, are rare tumors derived from thymic epithelium. TETs are heterogenous genetically and histopathologically.^1^, ^2^ Currently, the Masaoka stage or pathological TNM stage is considered the best predictor of the long‐term survival of TET patients; however, such values are confirmed only after surgery. In order to provide appropriate treatment, specific biomarkers that can predict the prognosis and therapeutic response before treatments are desired.

The survival is also determined by host‐related factors, including systemic inflammatory markers and the nutrition status. Recent studies have clarified that tumor‐related immune responses are significantly related to tumor progression.^3^ Cytokines produced by tumor cells or tumor microenvironment can stimulate the host inflammation, which leads to an increase in circulating peripheral blood cells, including neutrophils, lymphocytes, monocytes, and platelets.^4^ Since Virchow first noted leukocytes in neoplastic tissues and discussed the relationship between inflammation and cancers in 1881, the peripheral blood neutrophil‐to‐lymphocyte ratio (NLR), monocyte‐to‐lymphocyte ratio (MLR), and platelet‐to‐lymphocyte ratio (PLR) have been widely used to predict the prognosis of cancers, including gastric cancer, colorectal cancer, lung cancer, esophageal cancer, and breast cancer.^5^, ^6^, ^7^, ^8^, ^9^ Similarly, the prognostic nutritional index (PNI) proposed by Onodera et al. is a prognostic index that reflects both the nutritional and immunological statuses, and a low PNI has been reported to predict a poor survival in various types of cancer.^10^, ^11^, ^12^

Thus far, only a few studies have reported a relationship between systemic inflammatory markers or the nutrition status and the prognosis of TET patients. The present study therefore explored the prognostic value of the NLR, MLR, PLR, and PNI in 158 TET patients who underwent complete surgical resection.

## METHODS

### Study population

This retrospective study was approved by the Institutional Review Board at Nagoya City University Hospital (Nagoya, Japan) (approval number 70‐00‐0038). We reviewed the medical records of patients with TET, who underwent complete surgical resection from April 2004 to December 2019 at Nagoya City University Hospital. Patients were excluded according to the following criteria: (i) had recent steroid therapy, (ii) had active infection or other bone marrow disorders, (iii) had blood transfusion, (iv) had a treatment history of another type of cancer within one year, (v) underwent surgery for recurrence, (vi) had incomplete resection, and (vii) had incomplete clinicopathological or examination data or follow‐up information. We included patients with autoimmune diseases unless they had recent continuous steroid therapy.

### Definition of inflammatory markers and the survival

Data were compiled from individual patient medical case notes, electronic patient records, and pathology reports. Peripheral venous blood samples were collected within one month before the initiation of treatment. The report of a blood examination routinely performed was considered and the blood samples were not obtained prospectively. The NLR was derived by dividing the absolute neutrophil count by the absolute lymphocyte count in peripheral blood. The MLR and PLR were calculated in a similar manner. The PNI was calculated as 10 × serum albumin value (g/dl) + 0.005 × peripheral lymphocyte count (per mm^3^). To determine the optimal cutoff value for each inflammatory marker, a receiver‐operating characteristic (ROC) curve was generated to predict tumor recurrence. The maximum Youden index indicated the optimum cutoff. Disease‐free survival (DFS) was defined as the interval between the operation and the first incidence of detectable recurrence. The overall survival (OS) time was defined as the interval between the operation and death or the last follow‐up.

### Statistical analysis

The optimal cutoff values of the NLR, MLR, PLR, and PNI for the prediction of recurrence were determined with a ROC curve analysis according to the maximum Youden index. Survival curves were generated using the Kaplan–Meier method, and the log‐rank test was used to assess the statistical significance of differences between the two groups. A Cox proportional hazards model was used to estimate the hazard ratios and 95% confidence intervals. The prognostic variables identified by a univariate analysis were further analyzed in a multivariate Cox proportional hazards model. A two‐sided *p*‐value of <0.05 was considered to indicate a statistically significant difference. All of the statistical analyses in this study were performed using the JMP software program (version 12.0.1; SAS Institute).

## RESULTS

### Patient characteristics

A total of 158 TET patients who underwent complete resection were included in the study. The clinicopathological variables of the included patients are presented in Table 1. The study cohort included 84 men and 74 women, with a median age of 61 (range: 24–87) years old. Autoimmune disease was found in 27 patients, all of whom had thymoma. Eight patients who received preoperative steroid pulse therapy had thymoma. Two patients with thymic carcinoma and one with type B2 thymoma received preoperative chemotherapy, and one patient with thymic carcinoma received preoperative chemoradiotherapy. The histological subtypes of the 20 cases who received adjuvant therapy were as follows: type A thymoma, *n* = 1; type B1 thymoma, *n* = 1; type B2 thymoma, *n* = 4; type B3 thymoma, *n* = 2; thymic neuroendocrine tumor, n = 1; and thymic carcinoma, *n* = 11. The median follow‐up time was 61 (range, 0–174) months. The clinical courses of all patients were as follows: alive and well, *n* = 134; alive with disease, *n* = 14; died of other disease, *n* = 6; and died of disease, *n* = 4.

**TABLE 1:** Clinical and pathological findings in thymic epithelial tumor cases

Factor		Value	%
Age	Median	61	
Sex	Male	84	53.2
	Female	74	46.8
Tumor size (mm)	Median	47	
Histological type	Type A	12	7.6
	Type AB	35	22.2
	Type B1	35	22.2
	Type B2	43	27.2
	Type B3	13	8.2
	Thymic carcinoma	17	10.8
	Thymic neuroendocrine tumor	3	1.9
Autoimmune disease	Myasthenia gravis	22	13.9
	Pure red cell aplasia	3	1.9
	Good syndrome	2	1.3
	Rheumatoid arthritis	1	0.6
	None	130	82.3
Masaoka classification	I	48	30.4
	II	80	50.6
	III	22	13.9
	IVa and IVb	8	5.1

### ROC curves for the predictions of recurrence

**FIGURE 1:**
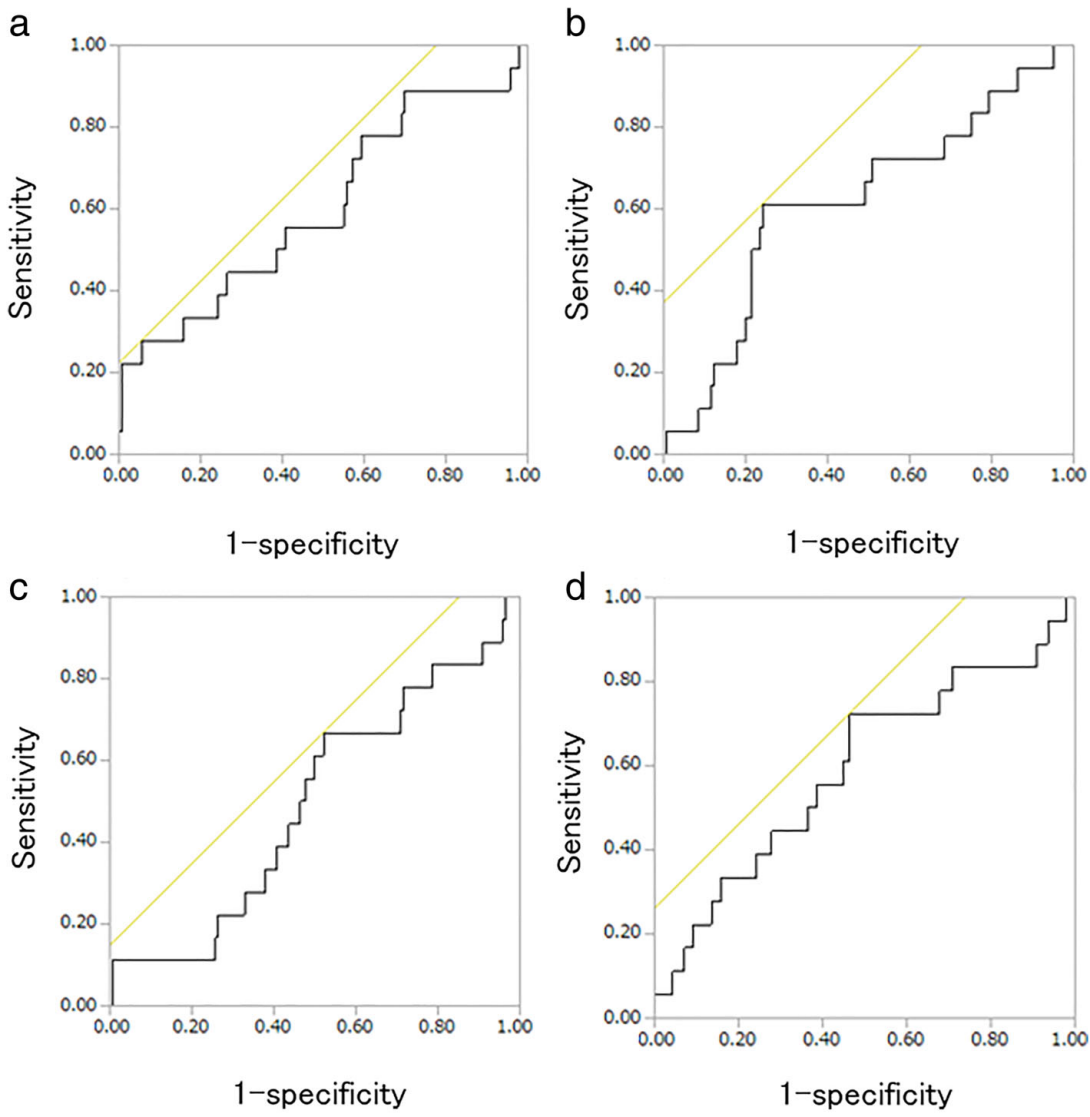
Receiver‐operating characteristic curve (ROC) analysis of (a) neutrophil‐lymphocyte ratio (NLR), (b) monocyte‐lymphocyte ratio (MLR), (c) platelet‐lymphocyte ratio (PLR), and (d) prognostic nutritional index (PNI) for disease‐free survival

In this study, we performed ROC analyses to select appropriate cutoff values for predicting recurrence for the inflammatory markers (Figure 1). The cutoff value was 4.35 for the preoperative NLR, with an area under the curve (AUC) of 0.604. The preoperative NLR had a sensitivity of 27.78% and a specificity of 94.29% for predicting recurrence. The best cutoff values for the preoperative PLR and MLR were 0.22 and 130.18, respectively, with AUCs of 0.618 (sensitivity = 61.11%, specificity = 75.71%) and 0.495 (sensitivity = 66.67, specificity = 47.86%). The cutoff value was 44.02 for the preoperative PNI, with an AUC of 0.591 (sensitivity = 72.22%, specificity = 53.57%).

### Inflammatory markers and the survival

**FIGURE 2:**
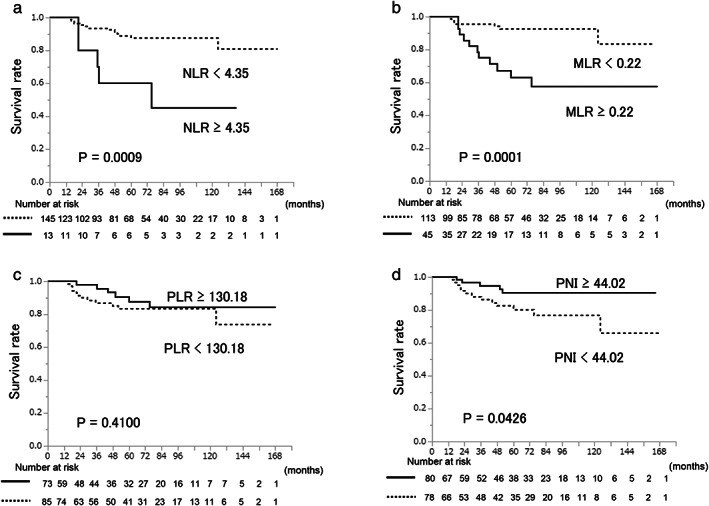
Kaplan–Meier curves for disease free survival according to the level of (a) neutrophil‐lymphocyte ratio (NLR), (b) monocyte‐lymphocyte ratio (MLR), (c) platelet‐lymphocyte ratio (PLR), and (d) prognostic nutritional index (PNI) [Correction added on 27 June 2022, after first online publication: figure 2 has been replaced.]

Kaplan–Meier curves demonstrated that a high NLR (≥4.35) (*p* = 0.0009), a high MLR (≥0.22) (*p* = 0.0001), and a low PNI (<44.02) (*p* = 0.0426) were associated with significantly lower DFS rates than a low NLR (<4.35), a low MLR (<0.22), and a high PNI (≥ 44.02), respectively (Figure 2). The PLR did not predict the DFS (*p* = 0.4100). The univariate analysis revealed that high‐grade malignant TET, including type B2 and B3 thymoma, thymic carcinoma, and thymic neuroendocrine tumor (hazard ratio [HR] = 8.0671, *p* = 0.0004); an advanced Masaoka stage (HR = 7.9015, *p* < 0.0001); a high NLR (HR = 4.8511, *p* = 0.0091); a high MLR (HR = 5.1991, *p* = 0.0006); and a low PNI (HR = 2.7766, *p* = 0.0398) were significant predictors of a poor DFS (Table 2). A subsequent multivariate analysis confirmed that an advanced Masaoka stage (HR = 5.5557, *p* = 0.0007) and a high MLR (HR = 3.3371, *p* = 0.0264) were independent predictors of a poor DFS (Table 2). No inflammatory markers predicted the OS after surgery (data not shown).

**TABLE 2:** A univariate and multivariate logistic regression analysis for clinical and pathological features and systemic inflammatory markers

	Univariate analysis			Multivariate analysis		
Factor	HR	95% CI	*p*‐value	HR	95% CI	*p*‐value
Age (≥60 years vs. <60 years)	1.4155	0.5556–3.7280	0.4647			
Sex (male vs. female)	0.5525	0.2032–1.4038	0.2133			
Tumor size (≥5 cm vs. <5 cm)	1.6798	0.6596–4.5756	0.2783			
WHO classification (type B2, B3, Carcinoma, neuroendocrine tumor vs. type A, AB, B1)	8.0671	2.2933–51.0351	0.0004	3.8573	0.9906–25.5180	0.0518
Masaoka stage (III or IV vs. I or II)	7.9015	3.0950–21.5740	<0.0001	5.5557	2.0733–15.9668	0.0007
NLR (≥4.35 vs. <4.35)	4.8511	1.5545–12.8849	0.0091	1.7290	0.5150–5.1011	0.3544
MLR (≥0.22 vs. <0.22)	5.1991	2.0454–14.1424	0.0006	3.3371	1.1553–9.8820	0.0264
PLR (≥130.18 vs. <130.18)	0.6643	0.2311–1.7120	0.4045			
PNI (<44.02 vs. ≥44.02)	2.7766	1.0469–8.6557	0.0398	1.4038	0.4926–4.6092	0.5366

Abbreviations: HR, hazard ratio; CI, confidence interval; NLR, neutrophil‐lymphocyte ratio; MLR, monocyte‐lymphocyte ratio; PLR, platelet‐lymphocyte ratio; PNI, prognostic nutritional index.

## DISCUSSION

In the present study, we explored the prognostic value of the pretreatment NLR, MLR, PLR and PNI in 158 patients with completely resected TETs. We found that, according to the univariate analysis, an advanced Masaoka stage, increased NLR, increased MLR, and decreased PNI were prognostic factors for a poor DFS. The subsequent multivariate analysis revealed that an advanced Masaoka stage and increased MLR were independent prognostic factors for recurrence.

The relationship between the NLR or MLR and prognosis appears to be complicated, and the precise mechanisms involved are not fully understood. An increased NLR or MLR indicates elevated neutrophils, elevated monocytes, or decreased lymphocytes. The systemic inflammatory response from cancer cells promotes the infiltration of neutrophils, which benefits cancer progression via the secretion of interleukin (IL)‐2, IL‐6, IL‐10, tumor necrosis factor α (TNF‐α), and vascular endothelial growth factor (VEGF).^13^ Neutrophilia as an inflammatory response inhibits the immune system by suppressing the cytokine activity of immune cells, such as lymphocytes, activated T cells, and natural killer cells.^14^, ^15^ Monocytes also play an important role in malignancies, as they interact with adaptive immunity by directing the recruitment and function of lymphocytes within the tumor microenvironment.^16^ Circulating monocytes take part in paracrine signaling and induce increases in the levels of many inflammatory cytokines and chemokines, including IL‐1, IL‐6, TNF‐α, and chemokine ligand 3.^17^ Lymphocytes also play an important role in malignancies. Lymphocytes serve a fundamental role in antitumor immunity.^18^ The increasing infiltration of tumors with lymphocytes is reportedly associated with an improved response to cytotoxic treatment and the prognosis in patients with cancer.^18^, ^19^

In the present study, we included 28 patients with autoimmune diseases who had not received recent continuous steroid therapy. All these patients were accompanied by thymoma. In fact, thymoma generate autoreactive T lymphocytes that are responsible for the development of associated autoimmune diseases.^20^, ^21^ Most of these T lymphocytes experience apoptosis before relapse in the systemic blood flow, thus patients with thymoma usually show normal peripheral lymphocyte count compared to healthy controls.^22^ However, there have been some studies reporting that sporadic patients affected by aggressive TETs show absolute peripheral polyclonal lymphocytosis.^23^, ^24^, ^25^ In the patients with autoimmune diseases, autoimmunity might affect the results.

In recent years, many studies have reported that a high NLR or MLR is associated with a poor survival in patients with various malignant tumors, including gastric cancer, colorectal cancer, lung cancer, esophageal cancer, and breast cancer.^5^, ^6^, ^7^, ^8^, ^9^ To our knowledge, there have been only five reports examining the association of the NLR with the prognosis of TETs, and the present one is the second‐largest study.^26^, ^27^, ^28^, ^29^, ^30^ The cutoff values used in those reports ranged from 1.96 to 4.10, and the value used in the present study was 4.3, which was similar to the values in the previous five reports. In all five reports, a high NLR was associated or tended to be associated with a poor prognosis. Our finding that a high NLR was associated with a shorter DFS supports these previous findings. Among those five reports, the one by Wang et al. described the relationship between the pretreatment MLR and prognosis. Those authors found that a high MLR was associated with tumor aggressiveness, and a co‐high maximum standardized uptake value (SUV_max_)/MLR was an independent risk factor for recurrence.^30^ In the present study, an increased pretreatment MLR was found to be an independent predictor of recurrence. There were 11 cases of recurrence with increased pretreatment MLR values, including five with local recurrence and six with dissemination. Among these 11 cases, three were Masaoka stage II, four were stage III, and the remaining four were stage IV. Four cases, including two stage II cases, one stage III case, and one stage IV case, had not received neoadjuvant or adjuvant therapy. We believe that patients with increased pretreatment MLR values might be eligible for neoadjuvant or adjuvant therapy, even if they are at an early Masaoka stage.

The PNI is an index reflecting the systemic immune‐nutritional status of patients. There is considerable evidence indicating that higher PNI values are associated with a better survival in malignancies.^31^, ^32^, ^33^ The PNI is initially designed to assess the perioperative nutritional status and predict perioperative complications.^10^, ^34^ While the serum albumin level is used to assess the nutritional status and immune function, a reduced albumin level is associated with tumor progression, metastasis, and increased risk of death after surgery.^35^ To our knowledge, this is the first report to describe the predictive value of the PNI in TET patients. The optimal cutoff value of the PNI to predict the prognosis of patients with TETs has not previously been investigated, so we conducted a ROC curve analysis to explore the optimal cutoff value of the PNI among TET patients. Our results were in line with those of previously published studies describing a significant relationship between the PNI and prognosis of cancers.

Several limitations associated with the present study warrant mention. First, this was a retrospective study conducted in a single institution, although it was a relatively large‐scale (*n* = 158) study to evaluate the prognostic value of pretreatment NLR, MLR, PLR, and PNI. It may not be possible to completely avoid selection and information bias. Further studies including more patients and long‐term follow‐up data from multiple centers are needed to validate the results. Second, we used the cutoff values determined based on ROC curves. At present, there are no established cutoff values. Future studies should endeavor to establish optimal cutoff values specific to TETs. Third, we used only the baseline values of the NLR, MLR, PLR, and PNI, rather than accounting for dynamic changes in each systemic inflammatory marker and nutrition status. In addition, various factors, including other disease conditions and medications, were not considered. Fourth, we found no significant relationship between the systemic inflammatory markers or nutrition status, including the NLR, MLR, PLR, and PNI, and the OS. In the present study, the median follow‐up time was 61 months. Patients with thymomas, which account for the majority of TETs, are expected to survive for a relatively long period of time. A much longer follow‐up period is needed to examine the association of the systemic inflammatory markers and the nutrition status with the OS.

In conclusion, our study showed that the MLR was an independent predictor of a poor DFS in patients with TETs who underwent complete resection. If our results are validated in future studies, the MLR may be recognized as a valuable biomarker for predicting the prognosis in TET patients.

## CONFLICT OF INTEREST

The authors have no conflicts of interest to declare.
